# Impact of Examiner Training and 3D Reconstruction on CBCT Detection of Anatomical Microstructure

**DOI:** 10.1590/0103-644020256910

**Published:** 2026-01-19

**Authors:** Julio Almeida Silva, Marina C Machado, Alvaro Cruz, Mike R Bueno, Daniel A Decurcio, Lucas R A Estrela, Yara Teresinha Corrêa Silva-Sousa, Carlos Estrela

**Affiliations:** 1 Department of Stomatologic Science, Federal University of Goiás, Goiânia, Brazil; 2 Department of Endodontics, Universidad de Guadalajara, Guadalajara, Jal, Mexico; 3 CROIF, Diagnostic Imaging Center, Cuiabá, MT, Brazil; 4 Graduate Program, Dental School, University of Ribeirão Preto, Ribeirão Preto, SP, Brazil; 5 Department of Preventive and Restorative Dentistry, São Paulo State University - UNESP, Araçatuba, Brazil

**Keywords:** Cone-beam computed tomography, anatomy, diagnosis, endodontics, 3D reconstruction

## Abstract

Cone-beam computed tomography (CBCT) has become a reference imaging method in dentistry, providing higher diagnostic accuracy and detailed visualization of complex internal anatomy. This study evaluated the impact of examiner training and three-dimensional (3D) reconstruction on CBCT detection of anatomical microstructures. CBCT scans obtained from 30 teeth with anatomical variations were evaluated by examiners across five skill levels. The same anatomical structures (root canals, isthmuses, and foramina) were assessed using dynamic navigation with multiplanar reconstruction (MPR) and a 3D reconstruction tool available in the e-Vol DX software. The experimental groups included the examiners: undergraduate student, undergraduate student with specific CBCT training, general practitioner, endodontist, and radiologist (control). Each examiner analyzed axial slices with and without 3D reconstruction, recording the number of structures identified and the influence of 3D assistance. Data were analyzed using Kolmogorov-Smirnov, Mann-Whitney, and Kruskal-Wallis tests (p < 0.05). For the general practitioner, 3D reconstruction significantly increased identification of root canals and foramina (p < 0.05), while other groups showed no significant differences between analyses. All examiners reported higher diagnostic confidence when using 3D reconstruction, with endodontists, general practitioners, and trained undergraduates indicating benefit in 100% of cases, and untrained undergraduates in 60%. The 3D reconstruction tool positively influenced the general practitioner's ability to detect root canals and foramina in mandibular premolars with complex anatomy. When using 3D reconstruction, examiners at all training levels achieved good diagnostic performance, enhancing both accuracy and confidence in anatomical interpretation.

## Resumo

A tomografia computadorizada de feixe cônico (TCFC) tornou-se um método de imagem de referência na odontologia, proporcionando maior acurácia diagnóstica e visualização detalhada de anatomias internas complexas. Este estudo avaliou o impacto do treinamento dos examinadores e da reconstrução tridimensional (3D) na detecção por meio da TCFC de microestruturas anatômicas em pré-molares inferiores com sulcos radiculares. Foram obtidas imagens de TCFC de 30 pré-molares inferiores com sulcos radiculares, avaliadas por examinadores com cinco níveis de habilidades. As mesmas estruturas anatômicas (canais radiculares, istmos e forames) foram analisadas utilizando navegação dinâmica com reconstrução multiplanar (MPR) e a ferramenta de reconstrução 3D disponível no software e-Vol DX. Os grupos experimentais incluíram os seguintes examinadores: estudante de graduação, estudante de graduação com treinamento específico em TCFC, cirurgião-dentista generalista, especialista em endodontia e radiologista (controle). Cada examinador analisou cortes axiais com e sem o uso da reconstrução 3D, registrando o número de estruturas identificadas e a influência da assistência 3D. Os dados foram analisados pelos testes de Kolmogorov-Smirnov, Mann-Whitney e Kruskal-Wallis (p < 0,05). Para o cirurgião-dentista generalista, o uso da reconstrução 3D aumentou significativamente a identificação dos canais radiculares e forames (p < 0,05), enquanto os demais grupos não apresentaram diferenças significativas entre as análises. Todos os examinadores relataram maior confiança diagnóstica ao utilizar a reconstrução 3D, sendo que o endodontista, o generalista e o estudante treinado relataram benefício em 100% dos casos, e o estudante sem treinamento em 60% dos casos. A ferramenta de reconstrução 3D influenciou positivamente a capacidade do cirurgião-dentista generalista em detectar canais radiculares e forames em pré-molares inferiores com anatomia complexa. Com o uso da reconstrução 3D, examinadores de todos os níveis de formação alcançaram um bom desempenho diagnóstico, aumentando tanto a acurácia quanto a confiança na interpretação anatômica.

**Figure:**
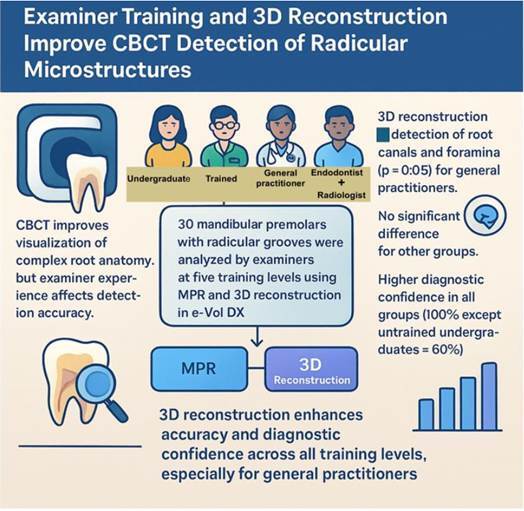

## Introduction

Periapical radiography is routinely used to assist in the diagnosis and planning of root canal treatments. However, as it provides only a two-dimensional representation of the root canal system’s complex anatomy, complementary imaging methods have been adopted to improve diagnostic accuracy, as well as for treatment planning^1^ ^,^ ^2^.

Cone-beam computed tomography (CBCT) represents a significant advancement in achieving precise diagnoses and selecting appropriate treatment plans. This technology allows detailed exploration of the region of interest through multiplanar reconstruction (MPR) ^3^ ^,^ ^4^ ^,^ ^5^ and three-dimensional imaging (cinematic rendering)(6). The use of CBCT imaging models provides a highly valuable methodological reference. The elimination of image superimposition across different planes (coronal, axial, sagittal, and oblique), combined with high resolution and contrast, distinguishes this imaging method from conventional techniques^3^ ^,^ ^4^ ^,^ ^5^ ^,^ ^6^. In addition to identifying the microanatomy of the root canal system, the use of CBCT scans has been recommended for detecting root cracks, fractures, perforations, resorptions, developmental anomalies, critical anatomical structures, and periapical lesions, among other important applications^3^ ^,^ ^4^ ^,^ ^5^ ^,^ ^16^.

There are several CBCT systems available, each equipped with specific software for navigating DICOM (Digital Imaging and Communications in Medicine) files, which enhances the versatility and applicability of this imaging technology^7^. These systems enable detailed visualization, manipulation, and measurement of anatomical structures, supporting more precise diagnostic and treatment decisions. Despite significant advances in diagnostic accuracy and treatment planning, CBCT still presents inherent challenges, particularly when high-density materials such as root canal fillings, metallic posts, or restorative materials are present, which may cause artifacts and compromise image interpretation^16^ ^,^ ^17^ ^,^ ^18^ ^,^ ^19^ ^,^ ^20^ ^,^ ^21^ ^,^ ^22^ ^,^ ^23^ ^,^ ^24^ ^,^ ^25^.

A post-processing software (e-Vol DX, CDT-Brazil, São José dos Campos, SP, Brazil) was developed to overcome current CBCT imaging limitations and to support the diagnosis, planning, and follow-up of complex endodontic cases^7^. This imaging tool provides high-resolution visualization through submillimetric voxel sizes, dynamic multiplanar navigation, and customizable volume parameters such as slice thickness, spacing, and image correction filters, as well as brightness and contrast adjustments. The main advantages of e-Vol DX over other software include full compatibility with all CBCT scanners capable of exporting DICOM data, an extended range for brightness and contrast adjustments, and flexible customization of slice thickness and sharpness, features often restricted or predefined in other applications. It also incorporates an advanced noise-reduction algorithm, predefined filters for root canal volume analysis, and the ability to magnify 3D reconstructions over 1000× without loss of resolution. This new CBCT software enhances image quality, supports decision-making in complex endodontic treatments, and contributes to more accurate diagnosis and improved treatment outcomes. The resulting image improvement promotes more rational prescription and interpretation of CBCT examinations ^7^ ^,^ ^8^ ^,^ ^9^ ^,^ ^10^ ^,^ ^11^ ^,^ ^13^ ^,^ ^14^ ^,^ ^15^.

Such tools play a critical role in assisting clinicians with the treatment planning of teeth presenting complex root canal anatomies^11^ ^,^ ^12^ ^,^ ^26^. Anatomical variations, such as isthmuses, lateral and accessory canals, apical deltas, and multiple foramina, pose significant challenges to effective cleaning, shaping, and obturation procedures. These intricate configurations often hinder the complete disinfection of the root canal system and the proper sealing of all pathways, thereby increasing the risk of persistent infection and endodontic failure. Advanced imaging modalities like CBCT allow clinicians to visualize better and understand these complexities, supporting more accurate diagnosis, improved case selection, specific clinical management for each case, and enhanced treatment outcomes^7^.

The morphological features of human permanent dentition can complicate root canal sanitization and obturation, increasing the risk of missed anatomy and treatment failure^26^ ^,^ ^27^ ^,^ ^28^. The use of specific CBCT filters in such cases enhances diagnostic accuracy by providing precise three-dimensional visualization and mapping of isthmuses, canal ramifications, and foramina, features that are critical for effective endodontic management and improved treatment outcomes^7^ ^,^ ^8^.

Significantly, diagnostic accuracy may depend on the examiner’s level of training. This study also investigates whether higher educational and professional experience levels are associated with interpretations that more closely approximate the reference standard, acknowledging that diagnostic performance often varies according to clinical experience^29^.

Therefore, the present study evaluated the impact of examiner training and 3D reconstruction on CBCT detection of teeth exhibiting distinct anatomical microstructure variations. The null hypotheses tested were:^1^ the level of professional training does not affect the interpretation of anatomical microstructures in CBCT scans; and^2^ the use of 3D reconstruction tools does not enhance examiner reliability in interpreting the internal anatomy of these teeth.

## Materials and methods

### Sample Selection

The study sample consisted of CBCT scans from 30 teeth exhibiting distinct anatomical variations, including isthmuses, accessory root canals, and accessory foramina. Mandibular premolars were selected according to inclusion criteria designed to ensure the presence and clear visualization of such anatomical microstructures. Teeth with extensive restorative materials, resorptive defects, or imaging artifacts that compromise the analysis were excluded. Each CBCT volume was carefully evaluated in axial, coronal, and sagittal planes to detect and classify the morphological variations within the root canal anatomy. This study was conducted following approval by the Institutional Ethics Committee.

### Image Acquisition

CBCT images were acquired using a Prexion 3D Elite 13-bit scanner (Prexion Inc., San Mateo, CA, USA) set to an isotropic voxel size of 0.100 mm, with a 52 mm height and 56 mm diameter field of view (FOV). The scans were obtained over 33.5 seconds (512 exposures per acquisition) using a tube voltage of 90 kVp, a current of 4 mA, a focal spot size of 0.20 × 0.20 mm, and total beam filtration > 2.5 mm eq. Al.

### Image Analysis

DICOM files were analyzed using e-Vol DX software (CDT Software, São José dos Campos, SP, Brazil) installed on a PC workstation (Windows 10, Microsoft Corporation, Redmond, WA, USA) equipped with an Intel i7-8750 processor (4.1 GHz) and an NVIDIA GTX 1070 graphics card (8 GB RAM).

CBCT scans of all teeth (n=30) were independently evaluated by five examiners with varying levels of experience, focusing on the same anatomical structures (root canals, isthmuses, and foramina). Initial analysis was conducted by a radiologist (stand reference) with over 10 years of experience in CBCT technology, using multiplanar reconstruction (MPR) to navigate the axial, coronal, and sagittal planes. Dynamic MPR navigation allowed parallax correction by rotating the planes^30^, facilitating optimal evaluation of the anatomical structures of interest, specifically the number of canals, isthmuses, and foramina. Four additional examiners, representing varying academic backgrounds and tomography experience, were selected for the experimental groups:^1^ an undergraduate dental student with no tomography training;^2^ an undergraduate dental student with specific CBCT training;^3^ a general practitioner with no tomography training;^4^ an endodontist (specialist and master with over 10 years of experience) with CBCT training.

The examiners received training to standardize navigation and adjust calibration with 10% of the samples. Each examiner analyzed a series of axial slice images obtained using MPR. Initially, only two-dimensional (2D) axial slices were evaluated, with parallax correction performed along the long axis of each tooth. Axial slices 0.2mm thick were generated for the isthmus and canal regions, and a final 1mm slice at the apical third was included to enhance foramina visualization. These axial images were saved as JPEG files and compiled into PowerPoint presentations, allowing each examiner to navigate forward and backward through the sequences, simulating coronal-to-apical and apical-to-coronal navigation^26^.

### Evaluation with and without 3D reconstruction

**Figure 1:**
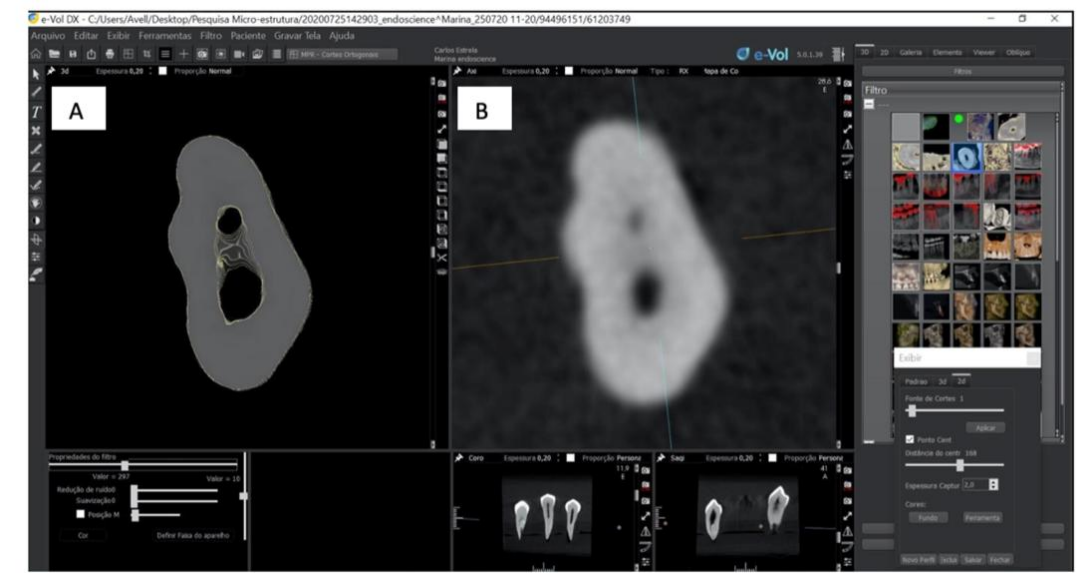
Visualization of 3D reconstructions using the e-Vol DX software (A) and axial slices (2D) (B).

After the first 2D evaluation, the identical axial sequences were processed using the 3D reconstruction tool in e-Vol DX software, maintaining the same orientation. Synchronization between the 2D and 3D images ensured matched density and scale, following the methodology previously described^10^ (Figure 1). Each examiner then re-evaluated the same structures (root canals, isthmuses, foramina) using the combined 2D axial slices and 3D reconstructions.

### Post-evaluation Feedback

After completing both evaluations, all examiners provided feedback on whether the 3D reconstruction tool enhanced their ability to identify the internal anatomical structures of mandibular premolars with radicular grooves. This feedback directly addressed the second null hypothesis by assessing whether the use of 3D reconstruction improves the examiners perceived diagnostic reliability.

### Statistical Analysis

Mean and standard deviation values for the number of isthmuses, root canals, and foramina were calculated for each examiner. Data normality was assessed using the Kolmogorov-Smirnov test. Comparisons between evaluations with and without 3D reconstruction were made using the Mann-Whitney U test, and differences between examiners and the control group were analyzed using the Kruskal-Wallis test. Statistical significance was set at p<0.05. Analyses were performed using SPSS software, version 20 (SPSS, Chicago, IL, USA).

## Results

The mean and standard deviation of the number of isthmuses, canals, and foramina identified by each group are shown in Table 1. When comparing the number of structures identified by the same examiner with and without the 3D reconstruction tool, a statistically significant difference (p<0.05) was found only in the General Practitioner group, where more canals and foramina were detected using 3D reconstruction. Specifically, the general practitioner identified an average of 2.70 ± 0.95 canals and 1.77 ± 0.77 foramina with 3D assistance, compared to 2.27 ± 0.69 canals and 1.37 ± 0.56 foramina without it.

For the other groups, the Endodontics Specialist, the trained undergraduate student, and the untrained undergraduate student, no statistically significant differences were observed between analyses performed with and without 3D reconstruction. Comparisons between the experimental groups (endodontics specialist, general practitioner, trained undergraduate, and untrained undergraduate) and the control group (radiologist using MPR navigation) revealed no statistically significant differences in the number of canals and foramina identified using the 3D reconstruction tool.**

**Table 1:** Mean and standard deviation of the number of isthmuses, canals, and foramina identified during the navigation of axial slices with and without the use of the 3D reconstruction tool.

	Isthmuses			Root Canals			Foraminas		
	Without 3D	With 3D	*p*	Without 3D	With 3D	*p*	Without 3D	With 3D	*p*
	***X* ± *S***	***X* ± *S***		***X* ± *S***	***X* ± *S***		***X* ± *S***	***X* ± *S***	
Endodontics Master	0.97±0.61^a X^	0.67±0.66^b X^	0.065*	2.43±0.82^a,b X^	2.73±0.91^a X^	0.152*	1.83±0.83^a,b X^	1.90±0.88ª ^X^	0.813*
General Practitioner	1.10±0.61^a X^	1.37±0.76^a X^	0.166*	2.27±0.69^a X^	2.70±0.95^a Y^	0.035*	1.37±0.56^a X^	1.77±0.77ª ^Y^	0.029*
Undergraduate Student with training	0.87±0.63^a,b X^	1.17±0.59ª ^X^	0.063*	2.37±0.76 ^a,b X^	2.50±0.82^a X^	0.488*	1.77±0.77^a,b X^	1.90±0.84ª ^X^	0.553*
Undergraduate Student without training	0.50±0.68^b X^	0.37±0.61^b X^	0.413*	2.57±0.86 ^a,b X^	2.53±0.86^a X^	0.956*	1.87±0.86^a,b X^	1.93±0.83ª ^X^	0.694*
Radiologist (Control)	0.63±0.67^a,b^	0.63±0.67^b^		3.00±1.02^b^	3.00±1.02^a^		2.53±1.20^b^	2.53±1.20ª	
p	0.002**	0.000**		0.017**	0.262**		0.001**	0.072**	

X: mean; S: standard deviation. *Mann-Whitney test - applied within rows, comparing data without and with 3D reconstruction for each anatomical structure analyzed (isthmuses, canals, and foramina) in each Experimental Group. Identical letters “X” or “Y” within rows indicate no significant difference (p > 0.05); different letters “X” and “Y” within rows indicate a significant difference (p < 0.05). ** Kruskal-Wallis test - applied within columns, comparing the Experimental Groups and the Control Group. Identical letters “a,” “b,” or “c” within columns indicate no significant difference (p > 0.05); different letters “a,” “b,” and “c” within columns indicate a significant difference (p < 0.05).

All examiners from the experimental groups reported greater reliability in identifying anatomical structures when using the 3D reconstruction tool. Specifically: 1- the Endodontics Specialist, General Practitioner, and trained undergraduate student reported that the 3D tool aided their interpretation in 100% of cases; 2- the untrained undergraduate student reported that the 3D tool was helpful in 60% of cases.

**Figure 2:**
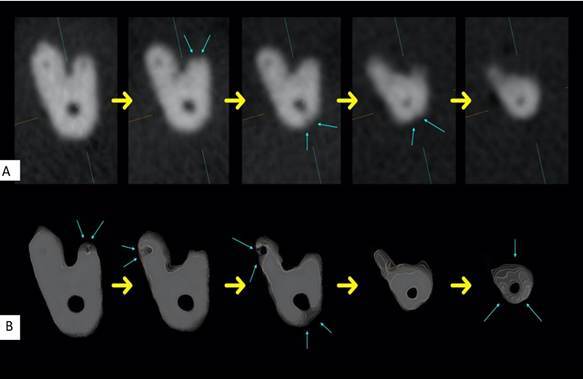
Comparison of the perception of the number of foramina in axial slices from the Experimental Groups obtained using MPR reconstruction. **(A)** Sequence of five axial slices in 2D images. **(B)** Sequence of five axial slices in 3D reconstructed images of the same sample. Yellow arrows indicate the direction from the most cervical slice to the most apical. Blue arrows highlight the foramina identified in each slice. The 3D reconstruction enhances foramina visualization, providing a more accurate anatomical representation.

**Figure 3:**
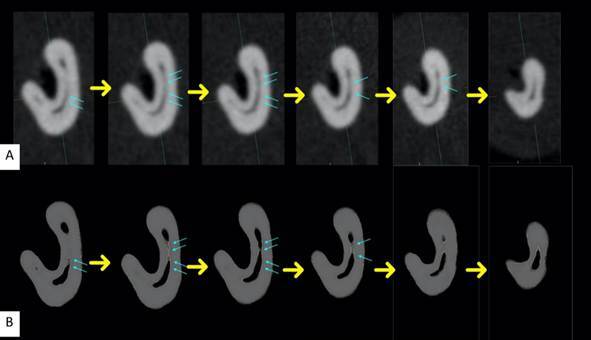
Comparison of the perception of the number of isthmuses in axial slices from the Experimental Groups obtained using MPR reconstruction. (A) Sequence of six axial slices in 2D images. (B) Sequence of six axial slices in 3D reconstructed images of the same sample. Yellow arrows indicate the direction from the most cervical slice to the most apical. Blue arrows highlight the isthmuses identified in each slice. The 3D reconstruction enhances isthmus visualization, providing a more accurate anatomical representation.

Figures 2 and 3 illustrate comparisons between the 2D axial slices obtained via MPR navigation and the corresponding 3D reconstructions, highlighting the enhanced visualization provided by the 3D images.

## Discussion

The use of 3D reconstruction tools significantly enhanced the identification of internal anatomical microstructures in mandibular premolars presenting with radicular grooves, with a particularly notable benefit observed among general practitioners. The statistically significant increase in the detection rates of root canals and foramina in this group underscores the diagnostic value of three-dimensional imaging in addressing the limitations inherent to conventional imaging modalities. These findings suggest that 3D reconstruction not only improves anatomical visualization but also contributes to more accurate and reliable endodontic assessments, especially for clinicians with less specialized training. The enhancement of diagnostic confidence reported across all examiner groups, irrespective of training level, underscores the educational value of 3D reconstruction. Its ability to elevate the performance of undergraduate students and non-specialists to levels comparable with trained radiologists demonstrates its potential as a powerful tool in radiologic education and clinical training. Therefore, both null hypotheses were rejected. Three-dimensional reconstruction proved to be a valuable tool in both education and clinical practice, particularly for professionals with less specialized training.

CBCT is a fundamental imaging modality that significantly enhances the accuracy of diagnosis, treatment planning, and follow-up in complex endodontic cases. The process of image post-processing and three-dimensional visualization depends heavily on specialized software^7^ that enables dynamic navigation and the application of indexing tools, thereby extracting clinically relevant information from volumetric data sets. Post-processing plays a pivotal role in elevating diagnostic quality, and several advanced techniques have been developed and widely applied to both computed tomography (CT) and magnetic resonance imaging (MRI) datasets. These include multiplanar reformations (MPR), maximum intensity projection (MIP), and volume rendering (VR). Among the latest innovations in 3D data visualization is the cinematic rendering reconstruction technique, which produces photorealistic three-dimensional images from conventional CT and MRI scans, offering unprecedented detail and depth perception^8^.

The methodology of the present study involved a comparison between multiplanar reconstruction (MPR) analysis and analysis using a photorealistic 3D reconstruction (7, 8). All imaging planes should be equally considered, although routine interpretation typically begins with MPR images. Additionally, general practitioners achieved a higher rate of anatomical structure identification when using 3D reconstruction. A distinguishing feature was the use of a dynamic navigation strategy to evaluate CBCT scans. Visualization through axial, sagittal, and coronal reconstructions provides optimal information regarding changes in anatomical microstructures. Dynamic navigation through the entire set of anatomical structures^9^, utilizing high-resolution CBCT imaging with a 0.1 mm voxel size and task-specific filters, designed to enhance image clarity and edge sharpness, as well as the possible use of cinematic rendering, improves image quality and increases the amount of diagnostic information that can be obtained^7^ ^,^ ^8^. A previous study^30^ used dynamic navigation along with a novel CBCT filter to analyze maxillary sinusitis of endodontic origin (MSEO) in posterior maxillary teeth. The approach allowed precise localization of root apices and sinus structures, revealing a high MSEO prevalence associated with root canal treatments, apical periodontitis, and apical proximity to the sinus floor. The integration of navigation and tailored imaging filters enhanced diagnostic accuracy for maxillary sinus alterations.

The complexity of root canal systems in mandibular premolars with radicular grooves has been documented in the literature^13^ ^,^ ^22^ ^,^ ^28^. Precise identification of anatomical variations, such as isthmuses, lateral canals, and apical ramifications, is critical for successful cleaning, shaping, and obturation. Undetected microstructures remain a common cause of endodontic failure, underscoring the importance of advanced imaging and meticulous exploration during treatment. Accessory root canals serve as strategic niches for microorganisms involved in root canal infections. Their detection using periapical radiographs is limited due to poor visibility and restricted access for endodontic instruments and antimicrobial agents. However, a significant number of accessory canals have diameters large enough to be visualized on high-resolution CBCT scans. Without specific diagnostic training, these structures can be easily overlooked or misinterpreted. A previously described methodology^10^ enables the identification of accessory canals using the e-Vol DX software in CBCT imaging. The technique involves navigating through ultra-thin slices (≤0.1 mm) in axial, coronal, and sagittal views. When a hypodense line suggestive of an accessory canal is observed, the MPR (multiplanar reconstruction) axes should be precisely realigned, parallel and perpendicular to the suspected structure, to correct for parallax. Once aligned, the accessory canal will typically appear as a "line-line-dot" pattern across the three orthogonal planes of the MPR view. This approach not only facilitates accurate identification of accessory canals but also allows for their visualization using volumetric rendering with task-specific filters. The method is simple to implement and offers valuable support in differentiating accessory canals from root fractures, thereby enhancing diagnostic accuracy in endodontic assessments.

Advanced imaging post-processing software has been shown to significantly improve image clarity, minimize artifacts, and enhance the visualization of fine anatomical details within the root canal system^7^ ^,^ ^8^. By utilizing high-resolution voxel settings, task-specific filters, and optimized rendering algorithms, e-Vol DX facilitates more accurate detection of complex structures such as accessory canals, isthmuses, apical deltas, and radicular grooves. These improvements contribute not only to diagnostic precision but also to more predictable clinical outcomes in endodontic treatment^7^ ^,^ ^8^ ^,^ ^9^ ^,^ ^10^ ^,^ ^11^ ^,^ ^12^ ^,^ ^13^ ^,^ ^14^ ^,^ ^15^.

Diagnostic and treatment-planning decisions among groups of dental surgeons with varying levels of experience and specialization were evaluated^31^. Enlarged periapical radiographs of 20 teeth, each linked to specific clinical scenarios, were assessed to identify the presence of periapical lesions and determine the appropriate clinical management. Seven distinct groups participated, each comprising 10 professionals representing various specialties involved in the decision-making process: general dental practitioners, final-year undergraduates, specialist trainees in restorative dentistry, consultants in restorative dentistry, specialist trainees in oral and maxillofacial surgery, consultants in oral and maxillofacial surgery, and specialist endodontists. The results showed that in 12 out of the 20 cases, interobserver agreement for radiographic analysis was rated as “excellent” or “good.” However, agreement on treatment decisions was lower, and the ranking of cases by diagnostic agreement did not correspond with that for treatment agreement. No case achieved “excellent” interobserver agreement for treatment decisions. Among the groups, endodontists demonstrated significantly higher agreement levels for both radiographic assessments and treatment decisions. Differences in training and specialization influence diagnostic and treatment decisions in endodontics, with endodontists showing the most incredible consistency in evaluations. In another study^32^ was compared decision-making among Turkish dentists with varying educational backgrounds. Eighty endodontically treated teeth were categorized into four equal groups based on symptoms and periapical lesions. Periapical radiographs and case details were distributed to participants, who included undergraduate students, general dentists, endodontic postgraduate students, and endodontists. For each tooth, participants selected one of five treatment options: no treatment (wait and see), non-surgical root canal treatment, apical surgery, combined retreatment and apical surgery, or extraction. Significant differences were observed among groups regarding preferred treatments. Postgraduate students and endodontists favored non-surgical retreatment more frequently in symptomatic and/or lesioned cases (groups 1, 2, and 3) compared to undergraduates and general dentists. In asymptomatic cases without lesions (group 4), all groups predominantly chose conservative management (“no treatment, wait and see”). The endodontists and postgraduate students tend to prefer more conservative treatments than less specialized practitioners. The findings of this study support existing evidence that specialization enhances diagnostic accuracy^32^. Moreover, the data indicate that technological advancements, such as 3D reconstruction, can help close the gap between clinicians with differing levels of training, thereby improving diagnostic outcomes across all groups.

The educational implications of these findings are particularly significant. Recent curricular reforms emphasizing the integration of digital technologies, a trend notably accelerated by the COVID-19 pandemic^33^, highlight the growing importance of incorporating advanced imaging tools into undergraduate dental education. This study underscores that, with targeted and adequate training, undergraduate students are capable of achieving diagnostic accuracy comparable to that of specialists when supported by state-of-the-art imaging resources. Such integration not only enhances students’ diagnostic competencies but also prepares them to effectively utilize evolving technologies in clinical practice, ultimately contributing to improved patient outcomes and fostering a more technology-savvy generation of dental professionals.

The impact of CBCT examinations performed according to European Commission guidelines on endodontic diagnosis was evaluated in a prospective observational study^34^. Fifty-three consecutive patients (81 teeth) from two endodontic specialist clinics in Sweden were followed. Following a comprehensive clinical examination, including patient history, clinical findings, and diagnostic tests such as intraoral radiography, a preliminary diagnosis was recorded before CBCT imaging. Subsequently, the same examiner reviewed the CBCT scans and established a post-CBCT diagnosis. Both pre- and post-CBCT diagnoses were documented at the patient and tooth levels. CBCT scans were acquired using standardized equipment and protocols across both clinics. Diagnostic changes occurred in at least one tooth in 22 patients (41%), affecting a total of 28 teeth (35%). These findings demonstrate that CBCT imaging, when applied according to European Commission guidelines, significantly influences diagnostic decision-making in endodontics. In our study, the incorporation of 3D reconstruction significantly enhanced diagnostic decision-making, especially in complex cases where conventional analysis might miss critical anatomical details. Since GPs perform the highest percentage of endodontic treatments on the population, their ability to predictably identify complex anatomical variants can improve the quality of their clinical endodontic care procedures.

Few dental studies have examined the use of 3D reconstruction techniques to create photorealistic CBCT images. These advanced visualization tools enhance image quality and provide more natural views of anatomical structures, improving differential diagnosis, clinical decision-making, and education, essential factors for clinical application. Incorporating 3D reconstruction in clinical practice boosts diagnostic accuracy and confidence, especially in complex cases requiring detailed anatomical understanding. Overall, the study highlights the value of 3D-enhanced CBCT in endodontics, advocating for its broader adoption to improve diagnostic consistency and treatment outcomes in teeth with complex anatomy, including radicular grooves.

## Conclusion

This study demonstrated that 3D reconstruction tools significantly enhance the interpretation of the internal anatomy of mandibular premolars with radicular grooves, particularly benefiting general practitioners. With the aid of 3D reconstruction, examiners across all levels of training achieved diagnostic accuracy in identified radicular grooves. These results confirm that both professional expertise and the integration of 3D reconstruction techniques play critical roles in the precise identification of anatomical structures in CBCT images. Incorporating 3D reconstruction into routine diagnostic protocols can therefore improve diagnostic reliability and support more effective treatment planning in cases involving complex internal anatomy.

## Data Availability

The research data are available upon request.
